# NONE OF THEM ARE REALLY FRIENDS: STAFF PERCEPTIONS OF THE SOCIAL WORLD OF PEOPLE LIVING WITH DEMENTIA IN LONG-TERM CARE

**DOI:** 10.1093/geroni/igac059.1224

**Published:** 2022-12-20

**Authors:** Kate de Medeiros

**Affiliations:** Miami University, Oxford, Ohio, United States

## Abstract

Nursing homes are charged with the care and protection of residents. Consequently, staff exercise tremendous control over residents’ day-to-day lives, including their social worlds. This is especially true for people living with dementia who may have challenges with communication. This paper examined how staff viewed friendships among dementia residents. Eleven staff members were asked to describe the friendship among the 20 residents in their charge to include who was friends with whom and what were the key features of the friendships. Results revealed that nearly all staff viewed residents as incapable of forming friendships among other residents. One exception was that many staff viewed felts that the male residents enjoyed a level of friendship that female residents did not. Overall, the findings point to the need to help staff move beyond a limited view of residents’ capabilities and discover ways to help foster a rich social environment

